# Text Mining Improves Prediction of Protein Functional Sites

**DOI:** 10.1371/journal.pone.0032171

**Published:** 2012-02-29

**Authors:** Karin M. Verspoor, Judith D. Cohn, Komandur E. Ravikumar, Michael E. Wall

**Affiliations:** 1 University of Colorado School of Medicine, Aurora, Colorado, United States of America; 2 Computer, Computational, and Statistical Sciences Division, Los Alamos National Laboratory, Los Alamos, New Mexico, United States of America; 3 Center for Nonlinear Studies, Los Alamos National Laboratory, Los Alamos, New Mexico, United States of America; University of Illinois-Chicago, United States of America

## Abstract

We present an approach that integrates protein structure analysis and text mining for protein functional site prediction, called LEAP-FS (Literature Enhanced Automated Prediction of Functional Sites). The structure analysis was carried out using Dynamics Perturbation Analysis (DPA), which predicts functional sites at control points where interactions greatly perturb protein vibrations. The text mining extracts mentions of residues in the literature, and predicts that residues mentioned are functionally important. We assessed the significance of each of these methods by analyzing their performance in finding known functional sites (specifically, small-molecule binding sites and catalytic sites) in about 100,000 publicly available protein structures. The DPA predictions recapitulated many of the functional site annotations and preferentially recovered binding sites annotated as biologically relevant vs. those annotated as potentially spurious. The text-based predictions were also substantially supported by the functional site annotations: compared to other residues, residues mentioned in text were roughly six times more likely to be found in a functional site. The overlap of predictions with annotations improved when the text-based and structure-based methods agreed. Our analysis also yielded new high-quality predictions of many functional site residues that were not catalogued in the curated data sources we inspected. We conclude that both DPA and text mining independently provide valuable high-throughput protein functional site predictions, and that integrating the two methods using LEAP-FS further improves the quality of these predictions.

## Introduction

There are now more than 75,000 experimentally determined structures in the Protein Data Bank (www.pdb.org [1]). Nearly 8,000 structures were deposited in 2010 alone, and the number of depositions per year is rising. In particular, the number from structural genomics initiatives recently cracked 10,000, and these include a large number of proteins with unknown function. A major challenge of modern structural biology is to fully realize the potential of this resource to advance drug development, e.g. to leverage structure determination of *Mycobacterium tuberculosis* proteins for structure-based drug design [2].

After obtaining an atomic structure of a potential target, the first key step in structure-based drug design is to identify functional sites that might directly mediate drug interactions [3]. Compounds that bind specifically to a target's active site can interfere with protein function, and such inhibitors are typically explored as drug leads. Unfortunately drug leads are unsuccessful when they inadequately block the active site, as often happens. To overcome this limitation, drug developers have begun targeting alternative sites where interactions can remotely disable protein activity; for example, a recently discovered inhibitor of HIV protease blocks a site that controls access to the active site [4]. Experimentally derived knowledge of such alternative sites is scarce, however, and computational methods are needed to identify both active sites and alternative, functionally important sites. In particular, allosteric sites, where molecular interactions can remotely control the behavior of the active site, represent a potentially large, untapped source of alternative sites for drug design [5].

There are a growing number of computational methods that aim to identify and characterize functionally important sites in protein structures for drug design (see, e.g. review [6]). We developed a method called Dynamics Perturbation Analysis (DPA), which uses analysis of protein dynamics [7], [8], [9], [10], [11], [12]. DPA exhibited good performance in detecting small-molecule binding sites in hundreds of proteins in a protein-ligand docking test set [8], [9], and is specifically designed to locate allosteric sites, where binding causes changes in protein structure and dynamics [9], [11]. The development of an accelerated approximate method called Fast DPA created the potential for high-throughput analysis of protein structures to predict functional sites using DPA [8]. Fast DPA enabled a typical protein domain to be analyzed in less than a minute using a single core of a desktop computer, bringing analysis of all ∼100,000 protein domains in version 1.75 of the SCOP database [13] within easy reach. Our preliminary application of DPA to ∼50,000 domains in an earlier version of SCOP confirmed the feasibility of this task [14].

The good performance of DPA on a controlled test set of hundreds of protein-ligand complexes suggested that DPA would be a valuable resource for structure-based drug design [8], [9]. In applying DPA to a comprehensive set of 100,000 publicly available protein structures, however, information to validate predictions is scarce compared to that for the test set. In most cases information about functional sites is simply not available. In the relatively few cases where information is available, we would like to use it for validation. However, even when the literature indicates that a small molecule binds to a protein at a specific site, this knowledge might not have made it into a database and it is therefore effectively hidden from a high-throughput computational system. For example, when proteins are crystallized without a known small molecule interaction, the interaction will be missing from a database of information derived from crystal structures. Indeed, one of the major problems in modern biology is the growing gap between the knowledge captured in the literature and what has been formalized in genomic databases [15]. Because it is simply not feasible to manually follow up on functional site predictions for 100,000 protein structures, it is necessary to develop automated methods for extracting relevant information from the literature.

There has been a recent increase in efforts to extract information from the biomedical literature automatically [16], [17], including protein-protein interactions (e.g. [18], [19]) and gene-disease relationships (e.g. [20], [21]). Text-based features have also been successfully integrated into models for classification of protein domains [22] as well as more generally of protein function prediction [23], [24]. The problem of text mining specifically for functional site information, however, has been largely underexplored. Because text describing functional sites involves mentions of specific residues, we wondered mentions of residues in text might be indicators of their functional importance, and therefore might provide supporting evidence for functional site predictions. Detection of residue mentions offers some interesting advantages as a text mining problem: not only do residue mentions exhibit regularities that can facilitate their detection [25], [26], [27], but they also can be independently validated using physical data (i.e., the protein sequence). Similar advantages were seen in extraction of point mutation information from text [28].

Here we demonstrate the integration of high-throughput structure-based and text-based functional site predictions. We applied DPA to a comprehensive set of ∼100,000 domains in the SCOP database. We found the predictions recapitulated much of the information about functional sites in the databases, but many of the predictions were left unvalidated. In parallel, we used text mining to automatically extract residue mentions from abstracts of papers about the protein structure. We provide evidence that residues mentioned in abstracts are often functionally important, and show that DPA sites containing residues mentioned in text are more likely to have validating information in the databases. Our results show that DPA predictions provide a valuable resource for drug design, and emphasize the importance of automated literature mining for protein function prediction. In particular, we found that integration of text analysis improves structure-based prediction of protein functional sites.

## Results and Discussion

### Protein structures and text corpus

The subject of our study was a comprehensive set *S* of 106,411 protein domains with publicly available, experimentally determined structures (Methods).

We performed structure-based function prediction on a subset *S_X_* of *S*, consisting of 98,934 domains with structures determined using X-ray crystallography. The importance of selecting just the X-ray structures was to create a set that was consistent in the type of structural model. To optimize compatibility with our structure-based prediction method, we only selected domains for which the entire structure was described using just a single chain identifier.

To perform text-based predictions, we compiled a corpus *C* from a set of MEDLINE abstracts linked to the domains in *S* (Methods). In all we retrieved 17,595 abstracts representing primary references for 30,816 PDB entries, covering 88,707 SCOP domains.

### Database-derived annotations of sites

We assembled a table of residues from the domains in *S* and annotated each residue with information corresponding to three types of database-derived data: whether the residue lies within a site near a small molecule in the protein structure (NSM); whether the NSM interaction has been validated by a manual literature search (NSM-valid); and whether the residue lies within a catalytic site identified by a manual literature search (CSA). We sometimes refer to the combined NSM-valid and CSA data as “curated annotations” as these are derived from manually curated functional site annotations.

**Figure 1:**
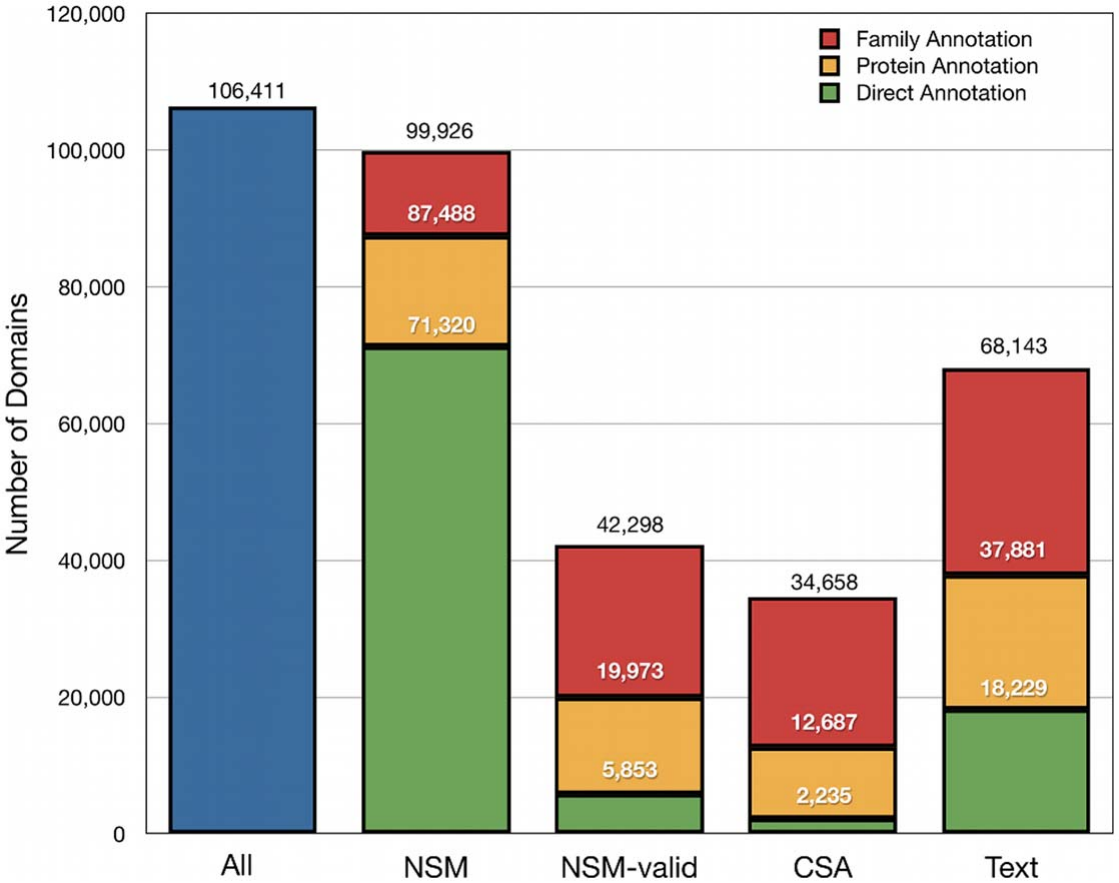
Availability of annotations for protein domains. Residues in protein domains were annotated using the following sources: 1) NSM, 2) NSM-valid, 3) CSA, and 4) text residue. A domain is labeled as annotated if one or more residues in the domain have an appropriate annotation. Stacked bars for each of the sources include cumulative numbers for direct annotations, annotations transferred at the protein level, and annotations transferred at the family level. The vertical order of the legend reflects the vertical order of the bars.

The coverage of *S* by these annotations is illustrated in Figure 1. To summarize, there were a total of 189,034 NSM sites in 71,320 of the SCOP domains. Using the BindingMOAD database of ligand interactions [29], [30] we identified 7,287 of these sites in 5,853 domains as valid (NSM-valid). The BindingMOAD database also provided an “invalid” annotation for 5,525 sites in 2,347 domains (NSM-invalid). Using the Catalytic Site Atlas (CSA) [31] we found catalytic site annotations for 2,235 of the SCOP domains.

#### Protein and family annotations

Multiple sequence alignments (MSAs) were used to transfer NSM, NSM-valid, and CSA annotations to residues at equivalent positions across SCOP families (Methods). Two types of transfers were performed, differing in their scope. Highly conservative *protein level* transfers were performed just between domains that correspond to the same protein. *Family level* transfers were performed between any two domains within the same SCOP family. The MSAs were also used to compute residue conservation scores, e.g., for identifying when annotations and predictions were associated with highly conserved residues (Methods).

Protein level transfers extended the number of domains in *S* having NSM annotations to 87,488, the number having NSM-valid annotations to 19,973, and the number having CSA annotations to 12,687 (Figure 1). Family level annotations increased the coverage even further, to 99,926 having NSM, 42,298 having NSM-valid, and 34,658 having CSA annotations (Figure 1). In terms of percentages, when annotations were transferred at the protein level, 82% of the SCOP domains had a NSM site, 19% had a NSM-valid site, and 12% were annotated with a CSA site. The percentages improved when annotations were rolled across the family: 94% of domains had a family-level NSM site, 40% had a family-level NSM-valid site and 33% had a family-level CSA site. Overall, the NSM, NSM-valid, and CSA annotations provided significant coverage of the SCOP domains; at the same time, however, there was a large gap between the percentage having a NSM site and the percentage having a NSM–valid site or CSA site, providing an opportunity for discovery of new supporting evidence from text.

### Structure-based predictions

#### Approach to structure-based prediction

We performed structure-based predictions using Dynamics Perturbation Analysis (DPA), as previously described [8], [9], [12]. Briefly, in DPA [9], a protein model is decorated with *M* surface points that interact with neighboring protein atoms. The probability distribution *P* ^(0)^(**x**) of protein conformations **x** is calculated in the absence of any surface points, and *M* protein conformational distributions *P* ^(*m*)^(**x**) are calculated for the protein interacting with each point *m*. Any model may be used to generate conformational distributions; for ease of computation, we used an anisotropic elastic network model [32], [33], [34], [35] of protein vibrations in contact with a temperature bath. The relative entropy *D* **_x_** between *P* ^(0)^(**x**) and *P* ^(*m*)^(**x**) is calculated for each point *m*, and is used as a measure of the change in the protein conformational distribution upon interacting with point *m*. Points with high *D* **_x_** values are selected and spatially clustered, and residues near these clusters are predicted to be functional sites. For high-throughput applications such as the present one, we developed an approximate Fast DPA method that performs as well as the original one in predicting small-molecule binding sites [8]. Further details of our implementation of DPA are provided in the Methods section.

#### Prediction statistics

We performed structure-based predictions on the subset *S_X_* of *S* that was best-suited for analysis by Fast DPA (hereafter referred to simply as DPA). As mentioned above, *S_X_* contained a total of 98,934 domains. A successful run produced two outputs: (1) a list of surface points with XYZ coordinates, a *D* **_x_** value, and a label for each distinct set of spatially clustered points meeting the threshold requirement; and (2) a table of DPA sites, each site consisting of a set of protein residues, where each residue has a heavy atom within 5 Å of at least one point from a given set of clustered points in (1).

DPA produced predictions for a subset *S_DPA_* covering 95,741 (97%) of the domains in *S_X_*. This yielded a set of predictions *P_DPA_* of 122,866 functional sites. Of all the domains in *S*, 90% have at least one functional site predicted by DPA. The coverage of those domains by DPA is therefore comparable to the coverage of the domains by family-level NSM annotations (94%), but is high compared to NSM-valid (40%) and CSA (33%) annotations. It is precisely this gap between the number of predictions and validated annotations that we wish to address by the literature analysis, seeking independent evidence supporting the predictions and increasing our confidence in them.

#### Significance of structure-based predictions

Several analyses highlighted the significance of DPA predictions. (1) Many of the NSM, NSM-valid, and CSA annotations were recovered by DPA. (2) Conservation of residues was highly enriched in DPA sites. (3) Many of the DPA sites corresponded to sites with a database annotation, and DPA sites with high conservation were enriched in annotations. We describe these analyses in turn, beginning with the coverage of annotations by DPA sites.

**Figure 2:**
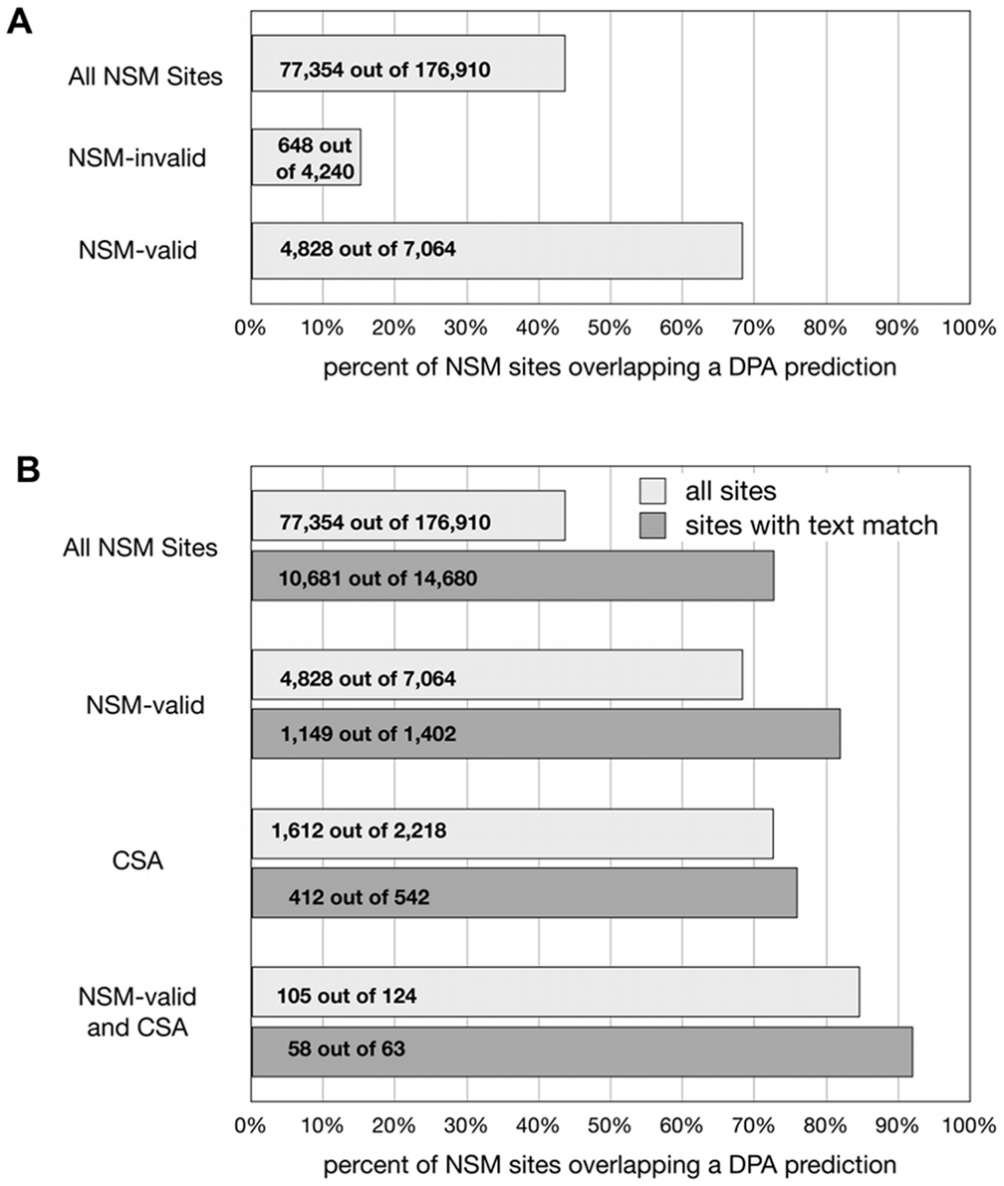
Coverage of sets of NSM sites by functional site predictions. (A) Coverage of NSM-valid sites by predictions is enriched and coverage of NSM-invalid sites is suppressed with respect to all NSM sites. (B) Sets of NSM sites with a text match are enriched in overlaps with predictions. Comparisons are made for all NSM sites, NSM-valid sites, NSM sites containing a CSA annotation, and NSM-valid sites containing a CSA annotation.

There were a total of 176,910 NSM sites among the domains in *S_DPA_* (Figure 2). The predictions overlapped 44% of these sites (Figure 2A). The overlap increased to 68% for sites that overlapped a NSM-valid site (Figure 2A), to 73% when the sites contained residues with direct CSA annotations (Figure 2B), and to 85% when the sites had both CSA and NSM-valid annotations (Figure 2B). Interestingly, the overlap decreased to 15% for NSM sites that were annotated as “invalid” by the MOAD database (Figure 2A). Thus, DPA preferentially highlighted functionally relevant NSM sites compared to all sites, and preferentially ignored NSM sites that were associated with irrelevant interactions.

Sequence conservation was substantially enriched in DPA predicted functional sites. Conservation MSAs were available to calculate lumped conservation scores for 62,759 of the sites in *P_DPA_* (Methods). We obtained a p-value for each of these lumped scores by calculating the probability of obtaining a score at least as good as what would be achieved by randomly selecting residues from the SCOP domain (Methods). For 14,778 (24%) of these sites, the p-value was < = 0.01, and for 8,983 (14%) of the sites, the p-value was < = 0.001 (Table 1). Thus, scores with a p-value< = 0.01 were found 24 times as often and scores with a p-value< = 0.001 were found 140 times as often as expected at random.

**Table 1:** Correspondence of DPA predictions to annotations.^a^

	Transfer MSA Available		Conservation MSA Available		Conservation P-value< = 0.01		Conservation P-value< = 0.001	
Number of DPA Sites	118397		62759		14778		8983	
**Direct Annotation**	52451	44.3%	29392	46.8%	11350	76.8%	7255	80.8%
NSM	50398	42.6%	28286	45.1%	10869	73.5%	6948	77.3%
Curated (NSM-valid or CSA)	5902	5.0%	3814	6.1%	2003	13.6%	1474	16.4%
Text	8527	7.2%	5211	8.3%	2687	18.2%	1803	20.1%
Curated or Text	13292	11.2%	8279	13.2%	4208	28.5%	2909	32.4%
Curated and Text	1137	1.0%	746	1.2%	482	3.3%	368	4.1%
**Protein-Level Annotation**	74527	62.9%	40815	65.0%	13108	88.7%	8245	91.8%
NSM	73417	62.0%	40313	64.2%	12966	87.7%	8153	90.8%
Curated (NSM-valid or CSA)	21714	18.3%	13410	21.4%	6285	42.5%	4552	50.7%
Text	22735	19.2%	14380	22.9%	7393	50.0%	4802	53.5%
Curated or Text	31291	26.4%	19402	30.9%	8845	59.9%	5733	63.8%
Curated and Text	13158	11.1%	8388	13.4%	4833	32.7%	3621	40.3%
**Family-Level Annotation**	100826	85.2%	59827	95.3%	14506	98.2%	8833	98.3%
NSM	100193	84.6%	59652	95.0%	14504	98.1%	8831	98.3%
Curated (NSM-valid or CSA)	40816	34.5%	28147	44.8%	11014	74.5%	7650	85.2%
Text	44990	38.0%	32110	51.2%	12210	82.6%	7853	87.4%
Curated or Text	54773	46.3%	37686	60.0%	12936	87.5%	8162	90.9%
Curated and Text	31033	26.2%	22571	36.0%	10288	69.6%	7341	81.7%

a Each entry in the table contains a number of DPA predictions that have a given type of annotation. Percentages are with respect to the numbers in the four subsets of DPA predictions indicated at the head of each of column. The subsets are increasingly restrictive from left to right: predictions in a domain for which a transfer MSA is available; predictions in a domain for which a conservation MSA is available; predictions for which the conservation P-value is no greater than 10^−2^; and predictions for which the conservation P-value is no greater than 10^−3^. Annotations are organized into three major sections that are increasingly expansive from top to bottom, as described in the text: direct annotations, protein-level annotations, and family-level annotations. The meaning of the NSM, NSM-valid, CSA, and Text annotations is as described in the text.

Coverage of DPA sites by curated annotations was significant, and the coverage became higher as the degree of residue conservation increased. Of all the predicted functional sites in *P_DPA_* for which a transfer alignment was available, 43% had a direct NSM annotation, and 85% had a family-level NSM annotation (Table 1). 5% had a direct and 35% had a family-level curated (NSM-valid or CSA) annotation. Among highly conserved DPA sites (p-value< = 0.01) 74% had a direct and 98% had a family-level NSM annotation. 14% had a direct and 75% had a family-level curated annotation. Among very highly conserved DPA sites (p-value< = 0.001) 77% had a direct and 98% had a family-level NSM annotation. 16% had a direct and 85% had a family-level curated annotation.

### Text-based predictions

#### Approach to text-based prediction

The fundamental assumption underlying our text mining approach is that an amino acid residue mentioned in an abstract from a publication about a protein structure is likely to be part of a functional site. We found support for this assumption in our analysis of abstracts associated with PDB structures.

To constrain the text mining to a relevant set of literature, we obtained the PubMed identifier for the primary reference for each PDB structure, where available. Each publication analyzed was therefore known to be relevant to a specific protein structure. We defined linguistic patterns corresponding to many possible variations for referring to residues in text. These patterns were applied to the abstract text and used to compile a database of residue mentions. Residue mentions that matched physical residues of the appropriate PDB entry were used as evidence that these residues are functionally important. Residue mentions in an abstract that could not be associated to a physical residue in the source PDB record associated with the abstract were ignored in subsequent processing.

Our text mining strategy was to extract from the text corpus all mentions of a specific residue, i.e., an amino acid at a specific position in the sequence (Methods). A simple mention of e.g. “Glycine” was not sufficient to support extraction; a localizable mention such as “Glycine 23” was required. We also included residues mentioned in the context of a mutation at a specific site, e.g., “Gly23Ala” (Methods).

We evaluated the performance of the residue mention extraction algorithm on three different corpora with manually curated residue mentions: *E_1_*, to evaluate extraction of mutation mentions from abstracts; *E_2_*, to evaluate extraction of residue or mutation mentions from abstracts; and *E_3_*, to evaluate extraction of residue or mutation mentions from full text. *E_1_* consisted of 813 abstracts containing mentions of mutations, compiled for MutationFinder [28] and divided into *E_1_dev_* development (305 abstracts) and *E_1_test_* test (508 abstracts) subsets. *E_2_* consisted of 100 abstracts containing mentions of residues and mutations, compiled and annotated by Nagel [36]. *E_3_* consisted of 50 full text articles containing mentions of residues and mutations, compiled and annotated by us for this study. All three of these corpora are available at http://bionlp-corpora.sourceforge.net/proteinresidue/. The full MutationFinder system is available at http://mutationfinder.sourceforge.net/.

**Figure 3:**
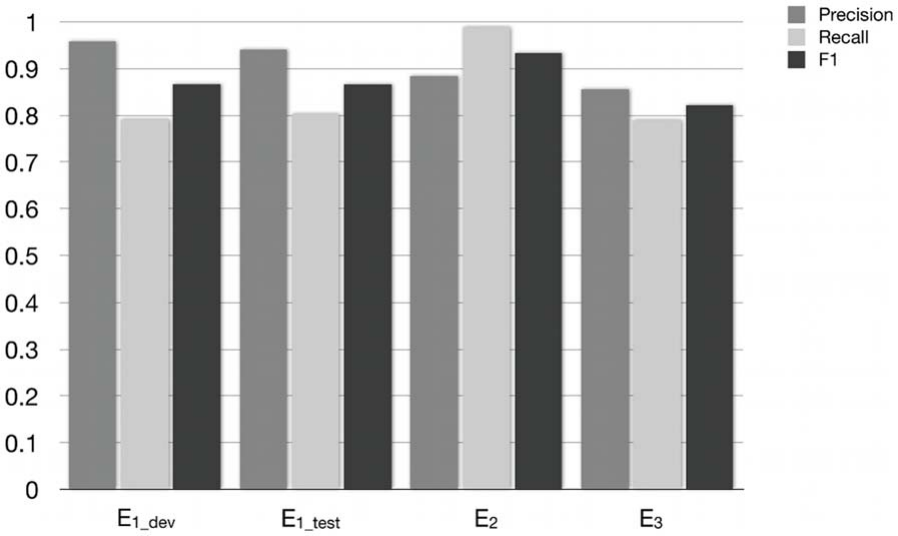
Intrinsic evaluation of the text mining system for extracting residue mentions and/or mutation mentions. Three corpora were evaluated. *E_1_* contains only annotations of mutations, and is subdivided into development (*E_1_dev_*) and test (*E_1_test_*) corpora. *E_2_* and *E_3_* include annotations of residues and mutations. *E_2_* consists of abstracts only, while *E_3_* consists of full text articles.

Results of the evaluation are summarized in Figure 3. There were 550 mutation mentions in the development set *E_1_dev_*. Our system extracted 454 point mutations, of which 435 were correct, resulting in a precision of 96% and a recall of 79% (see Methods for specific definitions of the precision and recall). In the test set *E_1_test_*, there were 907 mutation mentions. The system extracted 774, of which 728 were correct, leading to precision of 95% and a recall of 80%.

The curated mentions in *E_2_* include mentions of amino acids or mutations without corresponding sequence positions; here, we consider only 262 of the original 362 mentions, keeping only those that are tied to a specific position. Our system extracted 293 mentions, of which 259 were correct, resulting in 88% precision, 99% recall.

Finally, *E_3_* had 3120 curated residue mentions and mutation mentions. The system extracted 2875 mentions, 2463 were correct, leading to 86% precision and 79% recall.

The performance on these corpora indicates that the system effectively extracts residue mentions from abstracts and full text. Moreover, many of the errors could be understood by examining specific cases. For example, false positives in *E_1_* were predominantly due to the similarity of mutation expressions to the names of other entities such as cell lines (L23A), genes (E2F) and plasmids (R377). In *E_3_*, we found errors that were representative of previously noted broad differences between abstracts and full text publications [37]. For instance, mentions of residues and mutations were detected in the bibliography and other sections that contained tangential information. In some cases, citations in full text articles were confused with relevant mentions, e.g. “tyrosine (6)” was interpreted as a tyrosine at position 6. We also encountered residue mentions such as in the phrase “The catalytic residues Cys105L, His262L, and Asn286L together with Trp106L, Pro287L, and Trp288L …”. In the underlined cases, the system extracted the mention as a point mutation due to the association of the letter ‘L’ with the amino acid leucine. In actuality, in these cases the letter ‘L’ refers to the light chain of the protein and they are therefore single residue mentions rather than point mutations. This analysis of the false positives on the full text corpus indicates that the patterns originally developed for abstracts will need to be adjusted for full text articles. The system also missed some curated residue and mutation mentions because the patterns do not yet capture the full range of variations in how these mentions appear in the corpora. Overall, the system performed well enough to be useful for the present study, and analysis of specific cases pointed to opportunities for future improvement.

#### Validation of text residues using protein structures

**Figure 4:**
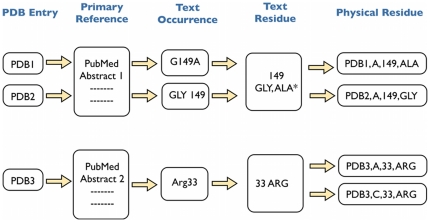
Illustration of the process for extracting residue mentions and mapping them to physical residues in protein structures. Text mining for residue mentions is performed on the abstracts from the primary references cited in the PDB. A text residue is represented by a residue number and one or more 3-letter amino acid codes. An amino acid for a text residue is marked with an asterisk if a text occurrence suggests it is a mutation from wild type. Physical residues corresponding to a text residue are indicated using a PDB ID, chain identifier, residue number, and 3-letter amino acid code.

A high level view of the process that builds on the extraction of residue mentions is shown in Figure 4, with the details available in Methods. The process aims to map residues mentioned in the text to physical residues in the relevant protein domains.

**Figure 5:**
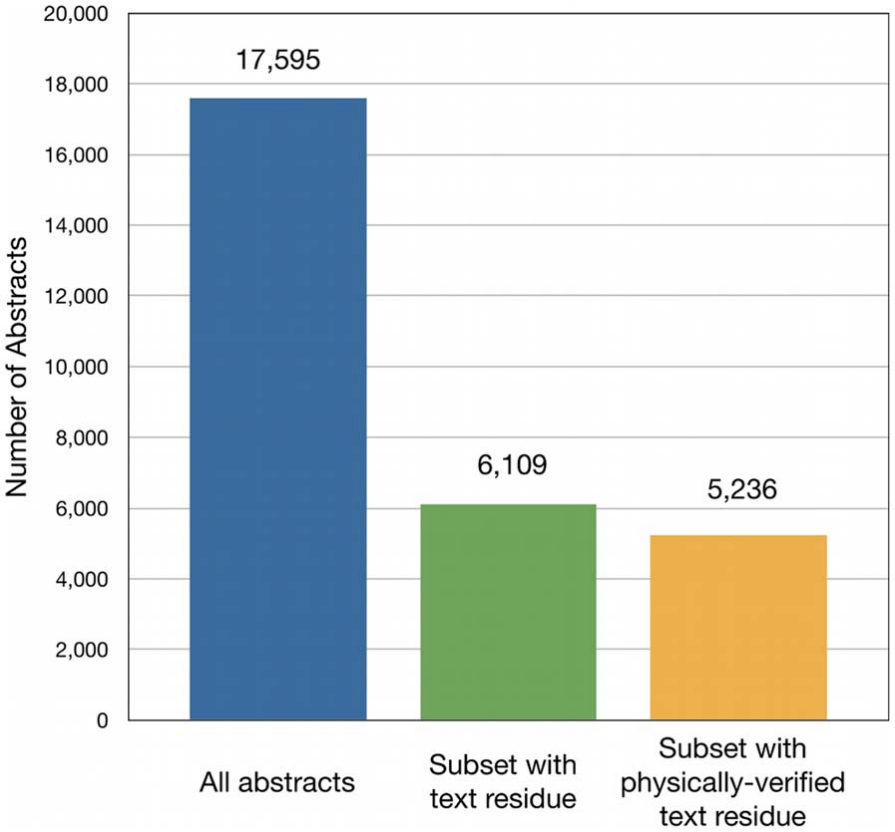
Availability of text-extracted residue mentions in the corpus of abstracts *C*. The number of abstracts with residue mentions is comparable when further constrained to those residues that can be mapped to physical residues in protein structures.

Text mining of the corpus *C* resulted in the identification of one or more text residues in 6,109 abstracts; 5,236 of these abstracts contained mentions of text residues that could be unambiguously matched to physical residues in relevant proteins, representing nearly 30% of the original abstracts (Figure 5). In all, 14% of abstracts with text occurrences were filtered out with a physical validation step; most of the filtered abstracts are expected to contain false positives.

A physically verified text residue was found for 18,229 of the domains in *S* (Figure 1). We transferred these text residue annotations to other domains at the protein level or family level using MSAs in a manner identical to that used to transfer NSM and CSA annotations. Transfers at the protein level increased the number of domains with a text residue to 37,881, and transfers at the family level further increased the number to 68,143. The resulting coverage of domains by text residues (64%) was significantly higher than the coverage by NSM-valid (40%) or CSA (33%).

#### Significance of text-based predictions

Residues mentioned in text were more likely to be found in a NSM-valid or CSA site than residues not mentioned in text. The subset of proteins that have at least one residue mentioned in text contained a total of 5,192,315 residues, 44,701 of which were mentioned in text. Of these, 17,457 (39%) matched a NSM-valid or CSA annotation. The remaining 5,147,614 residues did not have a text match. Of these, 310,821 (6%) matched a NSM-valid or CSA annotation. Thus, residues mentioned in text were 39/6 = 6.5-fold more likely to match a NSM-valid or CSA annotation.

The conservation of residues mentioned in text was also elevated relative to residues for which no mention was found. For residues with no matching text, the mean conservation score (fractional ranking of conservation compared to all residues in the same domain, 0 being lowest and 1 being highest) was 0.418 and the median was 0.356, while residues mentioned at least once in an abstract had a mean conservation score of 0.566 and a median of 0.531. This result lends additional support to the hypothesis that residues mentioned in text tend to be functionally important.

**Figure 6:**
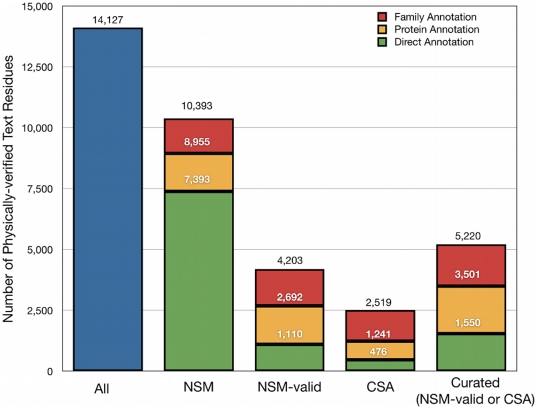
Overlap of physically-verified text residues with existing annotation sets. Annotation types and description of stacked bars for NSM, NSM-valid, CSA, and Curated is as described in Figure 1.

Finally, many of the residue mentions could be mapped to functional site annotations. The text mining of *C* yielded a total of 14,127 physically verified text residues (Figure 6). More than half of these (63%), could be mapped to a NSM site at the protein level. By contrast, relatively few could be mapped to a NSM-valid (19%) or CSA (9%) site. Matches were more frequent when including annotations transferred across the family: in this case, 74% of text residues could be mapped to NSM sites, 30% to NSM-valid sites, and 18% to CSA sites. At the same time, there were many more text residues that matched NSM sites than matched NSM-valid or CSA sites.

Overall, these results provide strong evidence for the functional significance of residue mentions. They also show that automatic extraction of text residues from the literature can identify a large number of small-molecule binding sites that are not already annotated in manually curated databases.

#### Limitations of the text mining approach

The corpus *C* consists of carefully selected articles that are manually provided as the primary reference for a given PDB record. This initial limitation was necessary to establish the fundamental viability of the LEAP-FS approach prior to generalizing the methods to a larger, noisier, set of literature. Many issues will complicate the text mining when we extend beyond this controlled set, including the need to ground residue mentions in a specific protein sequence, and ambiguity in protein naming. Indeed, we already have begun developing strategies both for extracting protein-residue relations [38] and for disambiguating protein mentions [39].

Another limitation is that we require an exact match to the position and (possibly mutated) amino acid in the protein sequence for all residue mentions. Because of this limitation, we are undoubtedly missing some valid matches. However, we expect such misses to be rare in the present study because each article is the primary reference for a deposited structure. We might catch more valid residue mentions by attempting to accommodate different residue numbering schemes, but this would almost certainly introduce some false positives, which we seek to minimize.

### Integration of structure-based and text-based predictions

Structure-based predictions were improved when integrated with residue mentions. Coverage of NSM sites by DPA sites increased substantially for NSM sites containing residues mentioned in text, from 44% without to 73% with text (Figure 2B). The availability of text evidence also increased the coverage of NSM-valid sites by DPA sites, from 68% without to 82% with text. The increase was not as significant when applied to the set of NSM sites containing a CSA annotation, from 73% to 76%, but was more significant when applied to the set of NSM-valid sites containing a CSA annotation, from 85% to 92% (though there are very few examples of the last case). Interestingly, the coverage of the set of NSM sites having a CSA annotation was the same as the coverage of the set of NSM sites matching a text residue (73%), and the coverage of the set of NSM-valid sites having a CSA annotation (85%) was similar to that of the set of NSM-valid sites matching a text residue (82%). The similarity of these percentages, combined with the lack of significant influence of text on the recall of NSM sites containing a CSA annotation, suggests the possibility that text mentions might be providing information that is similar to CSA annotations.

Structure-based predictions containing a residue mentioned in text were enriched in functional annotations (Table 1). Whereas 5% of all DPA sites had a curated (NSM-valid or CSA) annotation, 14% of the subset of DPA sites with a text residue had a curated annotation. The same trend was observed for curated annotations transferred using MSAs: in this case, 18% of all DPA sites had a protein-level annotation, compared to 58% of DPA sites with a text residue; 34% of all DPA sites had a family-level annotation, and 69% with a text residue had an annotation. Chances of finding a curated annotation for a DPA site therefore increased between two- and three-fold if the site contained a residue mentioned in text.

On the flip side, text residues that overlapped a DPA site were twice as likely to have functional annotations compared to ones that did not overlap a DPA site. Of the 14,127 physically verified text residues, 11,541 were in SCOP domains that had a DPA site. About half of these residues (5,509) overlapped a DPA site, and of these, 18%/39%/58% had a direct/protein/family level curated annotation. The other half (6,032) did not overlap a DPA site and only 9%/20%/30% of these had a direct/protein/family level curated annotation.

**Figure 7:**
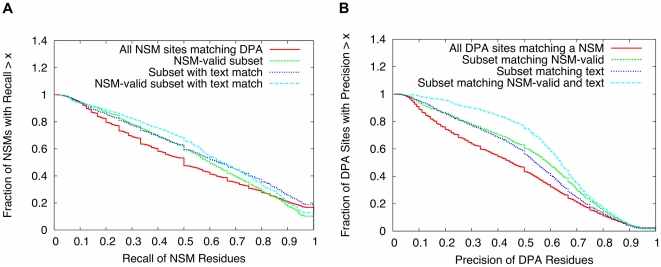
Residue-wise performance of functional site predictions. (A) Fraction of NSM sites satisfying various thresholds of recall. (B) Fraction of DPA predictions satisfying various thresholds of precision. The performance is compared using NSM vs. NSM-valid sites, and with or without text matches. The precise definitions of the recall and precision are described in the Methods.

Text evidence increased the overlap between residues associated with NSM sites and DPA predictions. The overlap was characterized using residue-wise recall and precision statistics (Methods), and was calculated for NSM vs. NSM-valid sites, with and without text evidence. Full results of the comparison are shown in Figure 7 and illustrate a clear trend of improvement in both recall (Figure 7A) and precision (Figure 7B) with the availability of validated NSMs and text. The main result of the comparison can be summarized by focusing on cases where the precision or recall is 0.5 or higher. Out of all NSM sites that match a prediction in *P_DPA_*, 53% of them had a recall of 0.5 or higher. The percentage increased to 63% for NSM sites containing a text residue, 62% for NSM-valid sites, and 69% for NSM-valid sites containing a text residue. 47% of all DPA sites had a precision of 0.5 or greater, increasing to 59% of DPA sites containing a text residue, 63% of DPA sites matching NSM-valid, and 77% of DPA sites containing a text residue and matching NSM-valid.

The above results show that text residues do indeed provide supporting evidence for structure-based predictions of functional sites. Moreover, text residues were often found for sites for which annotation is not yet available. Consider the set of 118,397 DPA sites in structures for which a MSA was available (Table 1). Direct curated annotations were found for 5% of these sites (Table 1, “Curated”), and text residues were found for 6% of the remaining sites for which no curated annotations were available (Table 1, subtract “Curated” from “Curated or Text”). Thus, use of text residues more than doubled the number of DPA site predictions for which supporting evidence was available. When annotations were transferred at the protein level (or family level), text annotations provided supporting evidence for 9,577 (or 13,957) sites that had no curated annotations. In addition, of the 14,127 physically verified text residues, just 1,550 of them were already associated with a direct, curated annotation, increasing to 5,520 at the family level of annotation (Figure 6).

Finally, a major product of our analysis is the full set of 8,720 DPA sites (with or without an MSA available) that include one or more matches to a physically verified text residue (File S2). A direct curated annotation was already available for 1,179 of these sites, and the remaining 7,541 represent our strongest leads for future functional site annotations. Many of these sites contain residues that can be transitively annotated by comparison to other proteins; however, in these sites there were a total of 6,640 DPA residues that have supporting literature evidence and yet had no direct or transitive annotation available. A further 5,663 of these residues could be linked to a non-curated or MOAD-invalid NSM interaction through a protein- or family-level MSA, leaving 977 residues that were solely identified using LEAP-FS integration of DPA and text analysis. These last predictions are entirely unique products of our method.

#### Significance of novel LEAP-FS predictions

To get a sense for the value of these predictions, we read the supporting text for a random sample of ten protein structures with little or no annotation information available (Table 2). Among these structures were 15 predicted residues that were mentioned in text: 2 residues that could be mapped to an unvalidated NSM site at the family level, 4 that could be mapped to a NSM-valid site at the family level, and 9 residues without any annotations at all. The text contained evidence for the possible functional importance of all of the residues, supporting our assumption that a residue mentioned in an abstract from a publication about a protein structure is likely to be part of a functional site. The supporting text exhibited variation in the type and strength of information provided, including evidence from mutation studies, sequence comparisons, and other sources. The residues were mostly associated with enzymatic activity (Table 2, last column), in agreement with our suggestion above that text mentions might be providing information that is similar to CSA annotations (Integration of structure-based and text-based predictions).

**Table 2:** Summary of text linked to residue mentions for a random sample of high-quality predictions.

PDB ID (Chain)	Protein Name	Residue(s)	Text summary
153L (A)	Goose lysozyme	Glu73^a^	NAG O6 directly hydrogen bonds to Glu73 OE2 [59]
1AB0 (A)	V32D/F57H Adipocyte Lipid-Binding Protein	Asp32, His57, and Cys117^b^	Phe57 in WT is part of the ligand binding portal, and Asp32 and His57 form a salt bridge in the mutant that controls access to the ligand binding site. Cys117 lies within the internal lipid-binding cavity [60]
1EYJ (A)	Fructose 1,6-Biphosphatase	Asp74^b^	Asp74 is involved in the forward reaction of this enzyme [61]
1GU9 (A)	Mtb alkhydro-peroxidase	Cys133, His137	Cys133 is a catalytic sulfhydryl group,His137 is involved in a relay to deprotonate Cys133 [62]
1I8B (B)	Chalcone synthase	Phe256^a^	Gly256 is in the active site in the wild type and the whole paper is about mutations to this residue, including G256F [63]
1LAR (A)	Receptor-like protein tyrosine phosphatase LAR	Leu1644	Leu1644 is responsible for inactivity of the protein and mutation to Tyr conferred robust PTPase activity to D2 domain [64]
1QOB (A)	Anabaena ferrodoxin	Gln70	Gln70 is at an interface that makes contact with a key interface but is not as critical as other residues in determining oxidation kinetics [65]
1TS2 (A)	Toxic shock syndrome toxin-1	His135, Gln136	H135A appears to reduce superantigenicity by altering properties of TCR interaction surface and is tied for the largest joint reduction in superantigenicity and lethality. Q136A causes a dramatic conformational change [66]
1XYH (A)	Human cyclophilin J	Gln52^b^	Gln52 expected to be an active site based on sequence alignment [67]
1YK3 (A)	Putative antibiotic resistance protein from Mtb	His130, Asp168^b^	His130 and Asp168 are both in the active site and have a putative roles in substrate binding and catalysis [40]

a Family-level NSM annotation available.

b Family-level NSM-valid annotation available.

**Figure 8:**
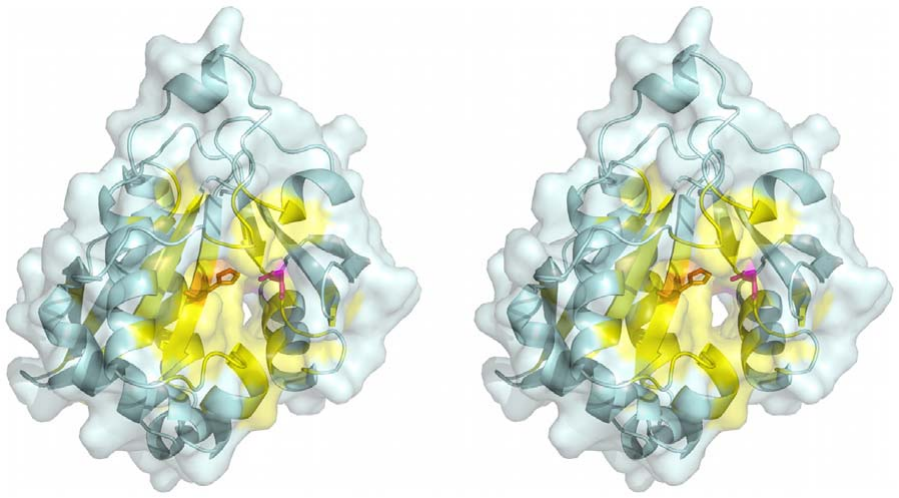
High-quality functional site prediction with text evidence for PDB entry 1YK3 [**40**]. The protein is displayed in wall-eyed stereo pairs as a light blue ribbon with a semitransparent surface. The predicted functional site from structure-based analysis is colored yellow, and the predicted residues mentioned in the abstract of the primary reference are rendered as sticks colored orange (His130) and magenta (Asp168).

To illustrate the kind of information that could be obtained in a more detailed read of the primary reference, we highlight one example, PDB entry 1YK3 [40]. Entry 1YK3 contains a structure of a protein from the *M. tuberculosis* structural genomics consortium which has been putatively identified as an acetyltransferase associated with antibiotic resistance. Structure-based prediction using DPA predicted a large functional site that included 54 residues: 17–20, 45, 65, 67–71, 73, 77, 81, 85, 90, 91, 96, 98, 103–109, 114–116, 129–133, 143–152, 166–170, 173, 176, 179, 180, 196, and 198 (Fig. 8). Automated analysis of the abstract highlighted the functional importance His130 and Asp168. There was no evidence for the importance of His130 found in any of the databases we examined. The Asp168 residue contacts a biologically relevant ligand in another structure in the same SCOP family. The full text of the primary reference, which was not automatically analyzed, states that His130 and Asp168 are likely catalytic residues in the putative active site [40]. The active site also includes many other predicted residues. In addition, a channel extending from the active site includes electron density that can be modeled as a crystallization detergent that contacts other DPA-predicted residues: Gly96, Trp98, Leu106, Ile133, Phe143, Leu147, and Ile151. A separate channel extending from the active site was suggested as a likely binding site for the acyl-CoA cofactor, but this channel is not obviously associated with the predictions. Overall the integrated LEAP-FS analysis highlighted a putative active site that might be worth mentioning in annotations, and suggested the possibility of a previously unappreciated functional role of the detergent-binding site, perhaps as an allosteric site.

Taken together, our data show the ability of LEAP-FS to highlight the functional importance of many residues not yet documented in biological databases. These results illustrate the potential for text analysis to make a substantial impact in providing supporting evidence for predictions, and in identifying new annotations.

### Related work

Our study investigated integration of structure analysis and literature analysis for improved predictions of protein functional sites. It is the first to quantitatively demonstrate improvement when integrating such methods; however, other approaches exist for functional site prediction (see, e.g., those listed in [3]), and these could also be potentially integrated with literature analysis. In particular, other structural analysis methods have been applied globally to publicly available protein structures, and, following our approach, these could be coupled to literature analysis. One specific example is the CASTp method which has been used to automatically map surface clefts to annotated functional sites in 4,922 PDB structures [41]. Another is the geometric potential method for discovering ligand-binding sites, which was applied to 5,263 protein chains in the PDB [42]. Many other structure-based functional site prediction methods exist (see, e.g., those listed in [12]) and some of these might be suitable for high-throughput analysis and be equally amenable to integration with the literature analysis.

Prior efforts have addressed information extraction from the protein structure literature, and we have drawn on these efforts where possible. The PASTA (Protein Activation Site Template Acquisition) system [43] aimed not only to recognize specific residue mentions, but also to explicitly relate those residues to a given protein and even to categorize the substructure of the protein where the residue is found using deep natural language processing techniques. Several systems addressing the more specific problem of extracting point mutations have appeared [28], [44], [45], [46], including MutationFinder [28], whose corpora we analyzed (corpus *E*_1_). These systems used regular expression patterns and one system additionally attempted to classify the functional impact of those mutations [47]. Many of these systems tackled the challenging task of recognizing protein mentions and normalizing them to a database identifier, a problem we deferred by constraining our literature to the set of abstracts directly linked to the PDB. Caporaso and colleagues [48] compared MutationFinder to a physical approach in which mutations were identified by aligning a PDB protein sequence with its UniProt counterpart and looking for differences.

Nagel and co-workers [36] adopted a text mining approach similar to ours to identify functional sites, and we analyzed a corpus from their study (corpus *E*_2_). They also aimed to extract from text the associated protein in a specific organism, a feature that we plan to integrate in future work. Some important preliminary steps were taken to combine this work with structure-based functional site prediction, but the results of this preliminary work were inconclusive [49].

Finally, it is worth noting that the curation process of the BindingMOAD database incorporates an information extraction step that employs a natural language processing tool called BUDA (Binding Unstructured Data Analysis) [50]. BUDA filters out literature that is unlikely to contain binding data and highlights binding information in the text (e.g. binding affinity data) for manual inclusion in the database. The database curators note that the BUDA tool is an important time-saver in their process, but that it cannot be used for fully automated database updates since the tool cannot adequately determine the precise protein-ligand pair in the crystal structure for which the affinity data is provided.

### Conclusions

The LEAP-FS approach combines two methods to achieve high-confidence protein functional site prediction. The first is a structure-based method known as Dynamics Perturbation Analysis (DPA) that predicts functional sites by considering the dynamics of physical interactions [9]. The second is a text mining method that extracts mentions of specific residues from PubMed abstracts. We found that each of the methods independently identified functionally important sites in proteins, and that predictions improved when the text-derived residues overlapped the DPA predicted residues. Moreover, text analysis provided completely new supporting evidence for many functional site predictions. We conclude that text analysis improves prediction of protein functional sites, and that it can have a substantial impact in high-throughput applications.

## Methods

See Table 3 for a glossary of terms and symbols used below and in the rest of the manuscript.

**Table 3:** Glossary of terms and symbols.

Term or Symbol	Meaning
NSM	Near a small molecule in the crystal structure
NSM-valid	Subset of NSM interactions annotated as valid in the MOAD database [26], [27]
NSM-invalid	Subset of NSM interactions annotated as invalid in the MOAD database [26], [27]
CSA	catalytic residue from the Catalytic Site Atlas [28] with evidence type LIT
Text Residue	physically-verified residue mentioned in abstract of primary PDB reference
Transfer MSA	Protein-level or family-level multiple sequence alignment used for transferring annotations between protein domains
Conservation MSA	Subset of family-level transfer MSAs involving ten or more distinct proteins, used to calculate residue conservation scores
*S*	Comprehensive set of 106,411 protein domains
*S_x_*	Subset of *S* consisting of 98,934 domains on which structure-based prediction was performed
*C*	Corpus of 17,595 MEDLINE abstracts representing primary references for PDB entries
*E_1_*	Evaluation corpus of 813 abstracts annotated with mutation mentions, compiled for MutationFinder
*E_1_dev_*	Development subset of E1, consisting of 305 abstracts
*E_1_test_*	Test subset of E1, consisting of 508 abstracts
*E_2_*	Evaluation corpus of 100 abstracts annotated with both amino acid residues and mutation mentions, compiled by Nagel et al. [33]
*E_3_*	Evaluation corpus of 50 full text articles containing mentions of residues and mutations, compiled by the authors for this study

### Protein structures

mmCIF files for 65,053 Protein Data Bank (PDB) entries were downloaded in early May 2010 [51]. Definitions of 110,800 protein domains were obtained from release 1.75 (June 2009) of the Structural Classification of Proteins (SCOP) database [13]. 106,411 of these domains fully corresponded to data found in 37,980 May 2010 PDB entries. The structure dataset *S* consists of these 106,411 domains (see File S1).

Data from mmCIF files were imported into an Oracle database using the openMMS toolkit version 1.5.1 [52]. Domain definitions were also imported into Oracle from the SCOP files dir.cla.scop.txt_1.75, dir.com.scop.txt_1.75, dir.des.scop.txt_1.75, and dir.hie.scop.txt_1.75., downloaded from http://scop.mrc-lmb.cam.ac.uk/scop/.

### Multiple sequence alignments for annotation transfer

For each of the 3,462 SCOP families containing at least two domains (covering 101,545 of the domains in *S*), we performed one or two levels of multiple sequence alignment (MSA) using MUSCLE version 3.7 with default settings [53]. The first MSA level was based on assigning each domain in a SCOP family a unique protein id based on database references from the struct_ref category of the mmCIF data. In order of preference, a domain was assigned the Uniprot entry name, Genbank id or PDB id of the mmCIF entity associated with the domain. For each of 3,300 families, we were able to perform a *protein-level* MSA for each protein ID with more than one domain in the family. These alignments, covering 96,137 domains, included all domains within a family associated with a particular protein ID. The second level of MSA was performed on a non-redundant set of domains within each family with at least two distinct protein IDs. This was accomplished by selecting the longest (or the first in SID alphabetical order if there was a tie) domain for each unique protein ID within a family. 2,320 families included at least two distinct protein sequence IDs, covering 87,057 domains in *S*. By merging the *protein-level* and the non-redundant *family-level* MSAs, we were able to generate a virtual alignment of all domains in the family by transferring *family-level* alignment positions from the non-redundant domains to their cohorts in the appropriate *protein-level* MSA.

### Annotation of protein residues

The three main types of annotation are NSM, NSM-valid, and CSA. The NSM-valid and CSA annotations together are sometimes referred to as “curated annotations” in the text.

#### Sites near a small molecule (NSM)

An NSM site was defined as the set of residues in a protein chain near one particular small molecule in a PDB entry. A protein chain was defined as an mmCIF chain associated with one or more SCOP domains; while a small molecule was defined very broadly as any mmCIF chain not associated with a SCOP domain. mmCIF chains in contrast to PDB author chains are comprised of a single molecule since they are not constrained by a one-letter chain id. By this definition, small molecules included simple organic molecules, ions, polysaccharides, short polypeptides, and nucleic acid chains. 91% of the 189,034 NSM sites found in *S* were associated with simple organic molecules or ions and thus had a small molecular weight compared to the SCOP domains. Only 2% of NSM sites were associated with polymers containing more than 20 residues and all of these were nucleic acid chains. “Near” was defined as a distance of 5 Å or less between a heavy atom in a protein chain residue and a heavy atom in a small molecule.

#### Validated sites near a small molecule (NSM-valid)

To annotate biologically relevant NSM sites, we used the Binding MOAD database (2009 version) [29], [30] to label a subset of the sites as valid (NSM-valid). The BindingMOAD database also provides “invalid” annotations for interactions that appear to be irrelevant; however, it is important to note that biologically relevant interactions can occur at sites of spurious interactions with crystallization additives and other irrelevant compounds, and therefore that an invalid NSM site might nevertheless be a functional site in some other context. Based on a manual reading of the primary references for PDB structures, the MOAD website provides the file every_bind.csv, which contains a listing of PDB id/ligand pairs annotated as either valid or invalid (among other data). The ligand in each pair is represented by MOAD as an ordered list of one or more 3-letter hetero codes. We matched the MOAD hetero code lists for a particular PDB id to the hetero codes (as ordered within the mmCIF file) for small molecules associated with NSM sites in that PDB structure. Thus, for example, PDB entry 13 gs is a glutathione S-transferase complexed with sulfasalazine. MOAD lists three ligands for 13 gs: sulfasalazine (SAS), glutathione (GLU-CYS-GLY), and MES [2-(N-morpholino)-ethanesulfonic acid]. The first two are labeled as valid ligand interactions and the third is invalid. The hetero code list for each of these ligands corresponds to the hetero code list of a small molecule for this PDB entry (glutathione is listed in the PDB as GGL-CYS-GLY); and multiple instances of these small molecules are near residues of the two glutathione S-transferase chains in the structure. Thus, depending upon which small molecule a glutathione S-transferase residue is near, it is labeled as NSM-valid or NSM-invalid.

#### Catalytic residues (CSA)

We obtained annotations of catalytic residues from the Catalytic Site Atlas version 2.2.12 [31]. The Catalytic Site Atlas database provides annotations with two evidence types: 1) LIT, which are derived by manual extraction from a curated literature corpus; and 2) PSI-BLAST, which comprise transitive annotations derived from PSI-BLAST comparisons to the sequences of structures with LIT annotations. To ensure consistency with our own transitive annotations, we only include the LIT annotations in our analysis. Annotated residues with evidence type LIT (CSA) were linked to PDB residues using the author chain id and author residue number.

#### Transitive annotation

Using the *protein-level* transfer MSAs, we were able to map annotation of residues in one domain to an equivalent position in all other domains from the same protein; while using the merged *protein-level* and *family-level* transfer MSAs, we were able to map annotation of residues in one domain to an equivalent position in all other domains within the same family. At both levels, annotations were transferred only in cases where the amino acids of both the reference and target domains were in the same “fuzzy” amino acid group, as defined on the Catalytic Site Atlas website (http://www.ebi.ac.uk/thornton-srv/databases/cgi-bin/CSA/CSA_Help.pl#templates) in the section on homologous entries.

### Dynamics Perturbation Analysis (DPA) of SCOP domains

Fast DPA [8] was performed on the subset *S_X_* of *S*, which consisted of 98,934 SCOP domains determined by X-ray crystallography and containing a single chain identifier. Given an input PDB structure, MSMS [54] was run with a 1.5 Å probe radius and a triangulation density of 1 vertex per Å^2^ to generate test points on the surface of the protein. The cutoff *r_c_* for interactions between protein C_α_ atoms was 10.5 Å. The cutoff *r_s_* for interactions between a test point and the protein was 15.5 Å, and the interaction strength between a test point and protein atoms was γ*_s_* = 12γ, or 12 times the strength of the interaction between two protein atoms. To predict functional sites, the distribution of *D* **_x_** values was fit using an extreme value distribution [8]. Points with *D* **_x_** values in the upper 96% of the distribution were selected and spatially clustered using the OPTICS algorithm [55] with a distance threshold of 5 Å and a minimum of 3 points per cluster. Any protein heavy atoms within 5 Å of any point in a cluster were selected and were used to define predicted functional sites. The sites were ranked according to the mean value of *D* **_x_** within the corresponding cluster of points; most SCOP domains had only one or two predicted sites, and differently ranked sites had similar rates of matching annotations. The analysis failed to produce predictions for 3,193 (3%) of the domains, for various reasons.

The median number of residues in a domain was 155 and the median time for a completed run (using a single core of an 8-core Mac Pro computer with 2×2.26 GHz quad-core Intel Xeon CPUs and 16 gb RAM) was 45 seconds. The longest run time was 89 minutes for a domain with 1,112 residues. Input to DPA was a PDB formatted file with a single record for each atom in the domain generated on the fly from the mmCIF data and the SCOP domain definitions in Oracle. When several possible sets of XYZ coordinates were available for an atom, the first one was selected to define the structure for analysis. Output from successful runs included: 1) a list of surface points evaluated by DPA, including XYZ coordinates, DPA chain ID (O, P, Q, R etc. for points which are part of a DPA predicted cluster, NN for points which are below the threshold, XX for points which above the threshold but do not cluster with enough points to form a cluster); 2) a list of protein residues in the SCOP domain with heavy atoms within 5 Å of a DPA cluster point.

### Residue conservation

#### Conservation of individual protein residues

Conservation scores were calculated for residues in the 54,819 SCOP domains from 323 families with non-redundant *family-level* MSA containing at least 10 sequences (called “conservation MSAs”). Each column in the non-redundant alignment was assigned the H.norm score calculated by the entropy function of the bio3d package for R [56]. H.norm is the normalized Shannon entropy for a 22-letter alphabet (20 amino acids plus a gap and a wildcard), where 1 indicates the most conserved columns (lowest entropy) and 0 the most diverse (highest entropy). Column scores from the non-redundant domains were then transferred to the rest of the family domains using the alignment positions derived from the virtual family alignment generated by merging the family and protein level MSAs.

#### Lumped conservation of DPA predicted functional sites

Each residue in a SCOP domain was ranked in descending order (i.e., highest conservation/lowest entropy first) using the H.norm value assigned to its position in the virtual SCOP family multiple sequence alignment. A lumped conservation score z for each DPA site was derived by multiplying together the percentile rankings of each of the L residues in a predicted site. A p-value, P, for this score was calculated by assuming a null model in which residues are drawn at random, yielding the following equation (derived for a similar purpose in Ref. [9]): 
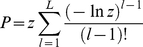
(1)


### Text corpus acquisition

To obtain relevant abstracts, we extracted PubMed IDs for primary references from mmCIF files of PDB entries linked to each domain in *S*.

We were able to extract the PMID for the primary reference of 31,367 out of the 37,980 PDB entries in our dataset. In the remaining cases, there was either no primary reference (47 cases), no PMID for the primary reference (3,775 cases) or our script was unable to extract a PMID (2,791). In addition, retrieval of 444 putative PMIDs representing 551 PDB entries returned empty documents.

Abstracts were retrieved from a local MEDLINE repository at UC Denver using the PMID as the search query [57]. This repository mirrors the PubMed search interface and is automatically updated nightly to stay in sync with PubMed.

### Residue mention extraction from text

We implemented our text mining system within the UIMA (Unstructured Information Management Architecture) framework [53], [58]. The system loads each document to be processed and applies a sequence of processing modules to detect both the basic amino acid residue mentions and the point mutation mentions, and then outputs the results in a standardized format which can straightforwardly be loaded into a relational database.

To find residue mentions in text, we defined linguistic patterns according to conventions for describing individual amino acid residues in PubMed abstracts. The patterns were designed to identify residue mentions that include both a specific amino acid name (either the full name, the 3-letter abbreviation, or the 1-letter abbreviation for the amino acid) and a particular position in the protein sequence. An example of a match to such a pattern is “Asp104” which corresponds to an aspartic acid amino acid residue at position 104 in the protein sequence. In addition to the standard abbreviations, the patterns also aim to normalize more variable linguistic expressions such as “cysteines at positions 6, 24, and 393”. Patterns for point mutations are designed to recognize mentions such as the standard short form “G146A” or the more linguistic “His-554 mutated to glutamine”.

The modules for detecting amino acid residues and point mutations are implemented as a set of Java regular expressions. These regular expressions are defined compositionally, in terms of sub-expressions corresponding to different components of a relevant mention. For instance, there exist expressions to detect single-letter and three-letter abbreviations of amino acids (e.g. “Asp”), as well as for the full names; there also exist expressions to detect positions (defined as a sequence of numbers, e.g. to match “104”) and expressions to define boundaries of the mentions and linguistic connectors. These sub-expressions are combined in various ways to arrive at the final expression patterns that are matched in the text (e.g. to enable matching the full residue mention “Asp104”). Our regular expressions were tweaked to appropriately accommodate for Unicode characters that can occur, especially in full text articles, e.g. hyphens that are encoded as an “em dash” character ‘—’ (Unicode character 2014), or arrows such as ‘→’ (Unicode character 2192). Finally, each detected mention was normalized to a representation consisting of the standard 3-letter (wild type) amino acid code plus the position; mutations had an additional column indicating the mutated amino acid residue.

For the intrinsic evaluation of the text mining system we reported on, we loaded the gold standard (manual) annotation from the appropriate sources into our UIMA framework and compared those loaded annotations with the system-generated annotations. We calculated Precision (P), Recall (R), and F1 score with the standard formulas, defined in terms of true positives (TP), false positives (FP), false negative (FN): 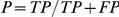, 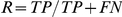, 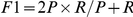. A true positive match to a normalized residue annotation requires agreement of the 3-letter amino acid code and the position of the residue. A true positive match to a normalized mutation annotation requires agreement of the 3-letter amino acid codes for both the wild type and the mutated residues, and the position of the mutation. A false positive occurs when the system produces a residue or mutation residue, but there is no matching normalized annotation in the gold standard. A false negative occurs when the gold standard includes an annotation that the system fails to produce. For the two corpora *E*_2_ and *E*_3_, a true positive match requires, in addition to a match of the normalized residue or mutation, a match of the start and end points (the annotation span) of the annotation with respect to the specific characters of the text. This span information was not available for *E*_1_ and therefore not considered in that evaluation.

### Association of text occurrences with physical residues

To associate text occurrences (residue mentions) with physical residues in PDB structures and thus with DPA predicted sites and other functional annotations used in our analyses, we processed the text collection in batch and collated all text residue mentions in a single data file, ordered by PMID and with each mention individually labeled as a basic amino acid mention or a mutation mention along with its normalized representation.

The process is summarized in Figure 4 and comprises the following steps:

Step 1. Group text occurrences for each abstract by site number. Each group is a text residue.
Step 2. Determine if the amino acid names for a particular text residue within an abstract are unambiguous - i.e. there is a single primary and, where applicable, one or more mutation amino acid names for the site. In 97% of cases where there are multiple text occurrences for the same site number within a single abstract, the primary amino acid name is unambiguous. Ambiguous text residues are eliminated from further analysis.
Step 3. Match text residues to physical residues in any protein chain of a PDB entry linked with the abstract by joining the site number to mmCIF auth_seq_id and either the primary or mutation (where applicable) amino acid names to mmCIF auth_comp_id.
Step 4. Group matching physical residues within a single PDB entry by text residue and determine if the matches are unambiguous. Multiple physical residues matching the same text residue within a single PDB entry are considered unambiguous if they are derived from a single molecular entity (as defined by the mmCIF label_entity_id). 98% of matches to physical residues are unambiguous. Ambiguous matches are eliminated from further analysis, as are any text residues that cannot be matched to a physical residue.

### Residue-wise recall and precision of DPA predictions

#### Recall of NSM residues

Let *R_NSM_* be the set of residues in an NSM site, and 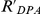 be the set of residues in all DPA predictions for the protein structure. The recall is defined as 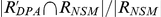, where 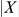 is the cardinality of set *X*.

#### Precision of DPA residues

Let *R_DPA_* be the set of residues in a DPA predicted site, 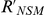 be the set of residues in all NSM sites for the protein structure, and 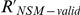 be the set of residues in all NSM-valid sites for the protein structure. The precision of DPA residues with respect to all NSM sites is defined as 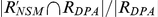, and the precision of DPA residues with respect to NSM-valid sites is defined as 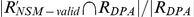.

## Supporting Information

File S1 **This file contains a list of the 106,411 domains that comprises the structure dataset** ***S*** **utilized in this study.** mmCIF files for 65,053 Protein Data Bank (PDB) entries were downloaded in early May 2010. Definitions of 110,800 protein domains were obtained from release 1.75 (June 2009) of the Structural Classification of Proteins (SCOP) database. 106,411 of these domains fully corresponded to data found in 37,980 May 2010 PDB entries. The file is tab-delimited and consists of 3 columns: (1) SID, the SCOP ID of the domain, (2) IS_SX, a binary flag indicating whether the domain is a member of the *S_X_* data set, that is, a subset *S_X_* of S, consisting of 98,934 domains with structures determined using X-ray crystallography and (3) IS_SDPA, a binary flag indicating whether the domain is also a member of the *S_DPA_* data set, that is the subset of *S_X_* for which DPA predictions exist.
(TXT)

File S2 **This file contains a listing of all the residues in 8,720 DPA predicted functional sites which include matches to one or more text residues extracted from the abstract of the primary reference for the PDB entry.** File S2 is a tab-delimited text file which, for the most part, contains one record per residue. However, where a residue is part of multiple DPA sites, it will appear in multiple file records. Columns in the file are: (1) DPA_CLUSTER_ID, unique identifier for DPA predicted site consisting of SCOP domain identifier concatenated with a period followed by a single letter from the set {O,P,Q,R,S,T} where O has highest average DPA score, P the second highest, etc.; (2) ENTITY_ID, mmCIF entity identifier (note: each distinct molecular entity within a PDB structure has a unique entity identifier for that entry); (3) CHAIN_ID, PDB author chain identifier; (4) RES_SEQ_NUM, PDB author residue sequence number; (5) ICODE, PDB insertion code; (6) RES_NAME, PDB author residue name (3-letter amino acid code); (7) IS_LIT_MATCH, binary flag indicating whether residue matches a text residue; (8) IS_CSA_LIT, binary flag indicating whether residue is listed in Catalytic Site Atlas with evidence code = LIT; (9) IS_NSM_VALID, binary flag indicating whether residue is near a small molecule in the PDB entry, which has been identified by MOAD as a valid protein-ligand interaction; (10) IS_NSM, binary flag indicating whether residue is near any small molecule in the PDB entry; (11) IS_CURATED_FAMILY, binary flag indicating whether residue is aligned with a curated annotation within the SCOP family, where curated comprises CSA-LIT or MOAD-valid annotation; (12) IS_NSM_FAMILY, binary flag indicating whether residue is aligned with any NSM site within the SCOP family; (13) NSM_HETERO_CODES, list of hetero codes for residues in nearby small molecules with MOAD status, if any, in parentheses (multiple small molecules are delimited by comma; DNA or RNA molecules are not broken down into hetero codes).
(TXT)
